# Analysis of survival factors after hepatic resection for colorectal cancer liver metastases: Does the R1 margin matter?

**DOI:** 10.3389/fsurg.2022.1020240

**Published:** 2023-01-06

**Authors:** Xiang-nan Ai, Ming Tao, Hang-yan Wang, Jing-lin Li, Tao Sun, Dian-rong Xiu

**Affiliations:** ^1^ Department of General Surgery, Peking University Third Hospital, Beijing, China; ^2^ Department of Hepatobiliary Surgery, Aerospace Center Hospital, Beijing, China

**Keywords:** colorectal cancer, liver metastasis, hepatectomy, R1 margin, BMI, CA19-9

## Abstract

**Introduction:**

The effect of liver margin on colorectal cancer liver metastases (CRLM) after hepatectomy has been controversial. In this study, we conducted a postoperative follow-up study of 205 patients with CRLM to clarify whether a positive margin is significant and to define the risk factors affecting CRLM survival.

**Methods:**

The data of 205 patients with CRLM who underwent surgical treatment at the Third Hospital of Peking University in the Department of General Surgery from January 2009 to December 2020 were retrospectively analyzed. The general data, surgical data and postoperative follow-up of the patients were statistically analyzed.

**Results:**

There were 130 cases (63.4%) of R0 resection and 75 cases (36.6%) of R1 resection. There were 136 males and 69 females, age 61 ± 11 years, and body mass index (BMI 24.5 ± 3.3 kg/m^2^). The overall survival rates at 1, 3, and 5 years for the entire cohort were 93.4%, 68.4%, and 45.5% in the R0 resection group vs. 93.2%, 53.7%, and 42% in the R1 resection group, respectively, which were not statistically significant (*P* = 0.520). The 1-, 3-, and 5-year disease-free survival rates of 63.2%, 33.3%, and 29.7% were significantly better in the R0 resection group than in the R1 resection group of 47.9%, 22.7%, and 17.7% (*P* = 0.016), respectively. After multivariable analysis, carbohydrate antigen 19-9 (CA19-9) > 39 U/ml (HR = 2.29, 95% CI: 1.39–3.79, *P* = 0.001), primary tumor perineural invasion (HR = 1.78, 95% CI: 1.01–3.13, *P* = 0.047), and BMI > 24 kg/m2 (HR = 1.75, 95% CI: 1.05–2.93, *P* = 0.033) were independently associated with poorer overall patient survival. The number of liver metastases >2 (HR = 1.65, 95% CI: 1.10–2.47, *P* = 0.016), the maximum diameter of metastases ≥50 mm (HR = 1.67, 95% CI: 1.06–2.64, *P* = 0.026), and vascular invasion of the primary tumor (HR = 1.65, 95% CI: 1.03–2.64, *P* = 0.038) were also independently associated with poorer disease-free survival.

**Conclusion:**

In patients undergoing hepatectomy for CRLM, the negative effect of the R1 margin should be downplayed, and although the disease-free survival of the R1 margin is shorter than that of the R0 margin, it has no impact on overall survival. To improve overall survival, extra attention should be given to the factors of preoperative BMI, preoperative CA19-9, and the presence of perineural invasion of the primary tumor.

## Introduction

Colorectal cancer (CRC) is the third most common malignant tumor in the world and has the second highest mortality rate (1). The liver is the most common site of CRC metastasis and liver metastases are one of the leading causes of death in CRC patients. Approximately half of CRC patients will develop liver metastases (2, 3). Liver metastases are detected at the time of diagnosis of colorectal cancer in approximately 20%–25% of patients, and in 40%–50% of patients, liver metastases are detected after radical colorectal cancer surgery (4, 5). In recent years, the survival rate of colorectal cancer liver metastasis (CRLM) has increased significantly due to the development of chemotherapeutic agents, targeted drugs and combination therapy (6). However, radical surgery remains the most critical method to achieve long-term survival in CRLM, with 5-year survival rates ranging from 40%–60% (2, 4, 7, 8). The liver margin is an important factor in determining whether radical treatment can be achieved. Over the years, knowledge of the R0 margin of the liver has gradually narrowed from 1 cm to ≥1 mm (9–12). Even though the R1 margin of the liver is now recognized as less than 1 mm, there are conflicting reports on whether the R1 margin is an independent risk factor for CRLM. The objective of this study was to clarify whether the R1 margin is significant and to define the risk factors affecting the survival of CRLM by conducting a postoperative follow-up study of 205 patients with CRLM.

## Materials and methods

### Patient selection

**Figure 1:**
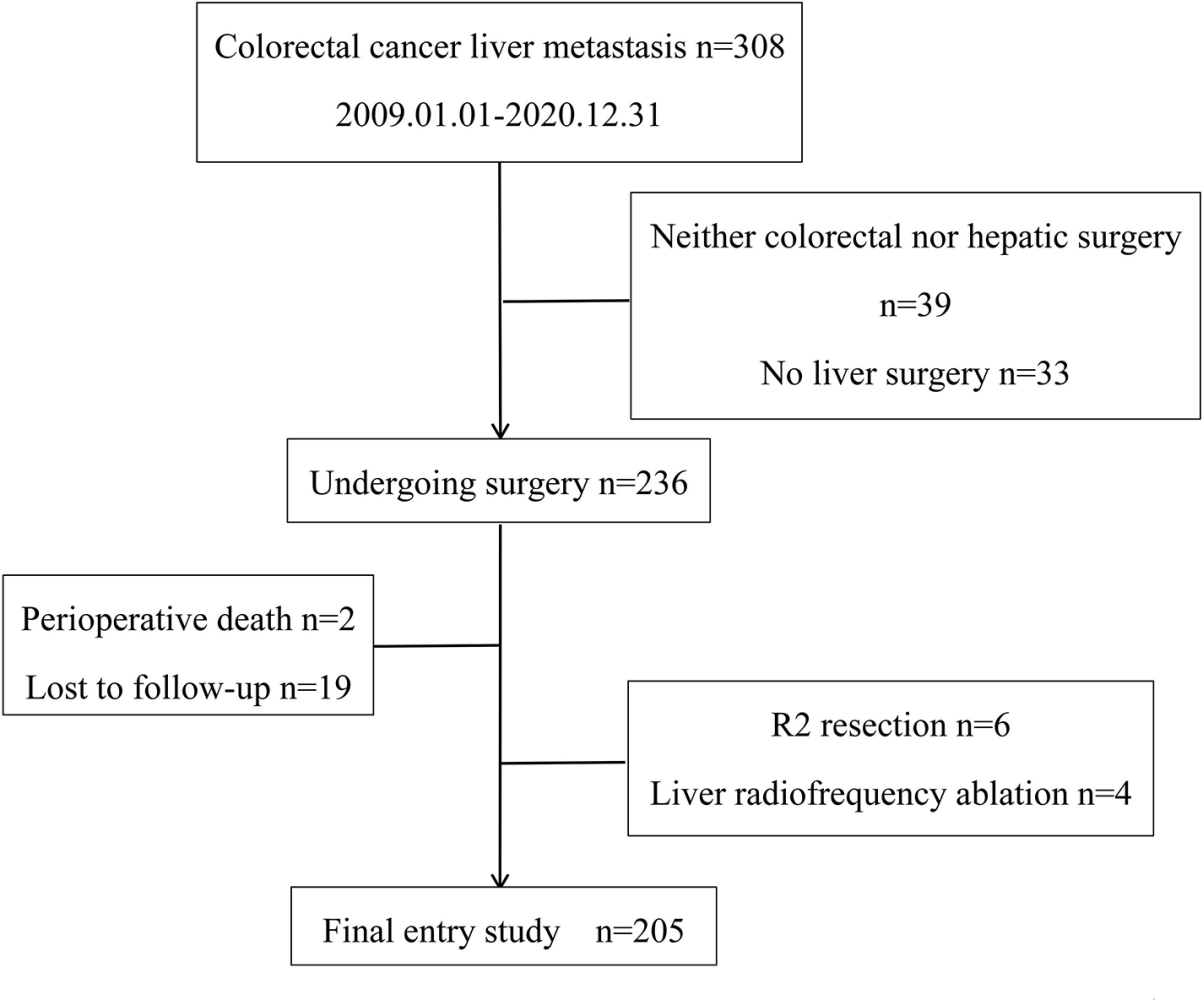
Patient inclusion and exclusion flow chart.

Patients with CRLM treated in the Department of General Surgery at the Third Hospital of Peking University from January 2009 to December 2020 were selected, and their clinical data were summarized. Inclusion Criteria: (1) Primary tumor is clearly colorectal cancer or clearly diagnosed as colorectal cancer by surgical pathology; (2) Complete medical record information; (3) Both colorectal and liver were treated surgically; (4) Patients were followed up regularly. Exclusion criteria: (1) Primary tumor or liver metastases are not resectable; (2) Palliative resection of the liver (R2 resection); (3) Liver undergoing radiofrequency ablation; (4) Liver metastases as recurrent lesions; (5) Perioperative death; (6) Incomplete clinical data; (7) None of the postoperative follow-ups were completed. The study was reviewed and approved by the Ethics Committee of Peking University Third Hospital and was conducted with the informed consent of the patients. A total of 308 patients with CRLM were treated at our center from January 2009 to December 2020, including 39 cases without surgery, 33 cases with colorectal surgery but without liver surgery, 6 cases with liver R2 resection, 4 cases with liver radiofrequency ablation, 2 cases with perioperative death, 19 cases with loss to follow-up, and 205 cases finally included in the study (Figure 1).

### Study design

Analysis of the effects of age, sex, BMI, primary tumor site, timing of hepatic metastasis (synchronous or metachronous), number of liver metastases, maximum diameter of metastases, carcinoembryonic antigen (CEA), CA19-9, neoadjuvant chemotherapy, postoperative adjuvant chemotherapy, American Society of Anesthesiologists (ASA score), surgery interval (staging or simultaneous surgery), operative time, intraoperative blood loss, type of hepatic resection (anatomical or nonanatomic resection), whether to transfuse blood, surgical approach (laparoscopy or open), T-stage of primary tumor, N-stage of primary tumor, primary tumor deposit, primary tumor vascular invasion, primary tumor perineural invasion, histological grading of primary tumor and liver margin on overall survival (OS) and disease-free survival (DFS) were performed, and the independent risk factors for OS and DFS were defined. The timing of hepatic metastasis was divided into synchronous liver metastasis and metachronous liver metastasis. Synchronous liver metastasis was defined as liver metastasis detected at the diagnosis of primary colorectal cancer or within six months after radical surgery for primary colorectal cancer (13), and metachronous liver metastasis was defined as liver metastases detected six months after radical surgery for primary colorectal cancer. Primary tumor deposits were defined according to the American Joint Committee on Cancer AJCC 8th edition staging as an isolated tumor nodule within the lymphatic drainage area of the primary tumor with no identifiable lymph nodes, blood vessels, or nerve structures within it. The resection margin refers to the distance from the tumor edge to the liver section. When there are multiple lesions, the closest distance is taken as the resection margin. R0 is defined as complete microscopic resection with margins ≥1 mm. R1 is defined as less than 1 mm from the resected surface of the liver under the microscope. For multiple lesions, R1 resection is defined as long as R1 is present in one lesion. OS was defined as the time interval between hepatectomy and death or the last follow-up visit. DFS was defined as the time interval between the time of hepatectomy and the first detection of recurrence or death. Recurrence is confirmed by reoperation pathology; if not operated, two or more imaging tests are required for diagnosis (enhanced computed tomography, enhanced magnetic resonance imaging, PET-CT examination).

### Follow-up protocol

All patients were reviewed every 3 months for 2 years after hepatectomy, including medical history, physical examination, tumor markers and imaging tests. Postoperative review every 6 months for 2–5 years. If patients fail to visit our center for follow-up examinations, they need to be followed up by telephone every 3–6 months to record their general condition, various review results and survival status.

### Statistical analysis

All statistical analyses were performed using Statistical Package for the Social Sciences (version 18.0 software), X-Tile (version 3.6.1 software), and R language (version 4.1.3 software). Continuous variables were expressed as (`x ± S) or M(Q1, Q3) and compared using independent samples *t* test or Mann‒Whitney *U* test. Categorical variables were expressed as cases and percentages and compared using the *x^2^* test or Fisher's exact test. For survival data, the Kaplan‒Meier method was used for description, survival curves were plotted, and the log-rank test was applied for comparison. For prognostic analysis, since it is easier to explain the results when converting continuous variables into categorical variables in clinical practice, X-Tile software was first applied to find the best cutoff values of continuous variables to be converted into categorical variables. Univariate Cox regression in R was subsequently applied to screen candidate influences, and then only variables with *p* values less than 0.05 and clinically significant variables were included in the multivariate Cox proportional risk model. The final independent risk factors affecting OS and DFS were identified by Cox regression multifactor analysis, and *p* values less than 0.05 were considered statistically significant. After multivariate analyses, a nomogram was constructed using the RMS package in R to visually predict the 1-year, 3-year, and 5-year OS rates for CRLM. The maximum value for each variable was set at 100 points. Calibration plots were used to determine whether the predicted survival rates were consistent with the actual survival rates. The nomogram was internally validated for discrimination and correction by 1,000 bootstrap resampling.

## Results

### Patient characteristics

The baseline characteristics of the 205 patients are shown in Table 1. There were 130 (63.4%) R0 resections and 75 (36.6%) R1 resections. There were 136 males and 69 females, age 61 ± 11 years, range 27–87 years, and BMI 24.5 ± 3.3 kg/m^2^.

**Table 1:** Demographic and clinical characteristics of 205 patients with colorectal cancer liver metastases.

Variables	All patients	R0	R1	*P* value
Number, %	205	130 (63.4)	75 (36.6)	
**Sex, *n*, %**				
Female	69 (33.7)	46 (35.4)	23 (30.7)	
Male	136 (66.3)	84 (64.6)	52 (69.3)	0.491
Age, years, mean ± SD	61.5 ± 11.1	60.9 ± 11.3	62.4 ± 10.8	0.342
BMI, kg/m^2^, mean ± SD	24.5 ± 3.3	24.4 ± 3.3	24.6 ± 3.4	0.658
**Surgery Interval, *n*, %**				
Staging surgery	73 (35.6)	49 (37.7)	24 (32)	
Simultaneous surgery	132 (64.4)	81 (62.3)	51 (68)	0.413
**ASA score, *n*, %**				
I	31 (15.1)	23 (17.7)	8 (10.7)	Ref
II	153 (74.6)	90 (69.2)	63 (84)	0.114
III	21 (10.3)	17 (13.1)	4 (5.3)	0.572
Operative time, min, mean ± SD	394.7 ± 165.2	373.4 ± 152.4	431.7 ± 180.5	**0** **.** **015**
**Primary site, *n*, %**				
Colon	129 (62.9)	78 (60)	51 (68)	
Rectum	76 (37.1)	52 (40)	24 (32)	0.254
**Timing of hepatic metastasis, *n*, %**				
Synchronous	147 (71.7)	92 (70.8)	55 (73.3)	
Metachronous	58 (28.3)	38 (29.2)	20 (26.7)	0.695
**No. of liver metastases, *n*, %**				
≤2	126 (61.5)	84 (64.6)	42 (56)	
>2	79 (38.5)	46 (35.4)	33 (44)	0.223
Size of largest tumour, mm, mean ± SD	33 ± 24	31 ± 22	37 ± 27	0.086
**Preoperative CEA level, *n*, %**				
≤5 U/ml	56 (27.3)	38 (29.2)	18 (24)	
>5 U/ml	149 (72.7)	92 (70.8)	57 (76)	0.419
**Preoperative CA19-9 level, *n*, %**				
≤39 U/ml	121 (59)	74 (56.9)	47 (62.7)	
>39 U/ml	84 (41)	56 (43.1)	28 (37.3)	0.421
**Neoadjuvant chemotherapy, *n*, %**				
No	116 (56.6)	82 (63.1)	34 (45.3)	
Yes	89 (43.4)	48 (36.9)	41 (56.7)	**0**.**014**
Blood loss, ml, M (Q1, Q3)	450 (200, 900)	400 (122, 800)	500 (200, 1100)	**0**.**023**
**Type of hepatic resection, *n*, %**				
Anatomical	40 (19.5)	32 (24.6)	8 (10.7)	Ref
Anatomical + Wedge	47 (22.9)	24 (18.5)	23 (30.7)	**0**.**006**
Wedge	118 (57.6)	74 (56.9)	44 (58.6)	**0**.**048**
**Transfusion, *n*, %**				
No	121 (59)	78 (60)	43 (57.3)	
Yes	84 (41)	52 (40)	32 (42.7)	0.708
**Postoperative chemotherapy, *n*, %**				
No	13 (6.3)	6 (4.6)	7 (9.3)	
Yes	192 (93.7)	124 (95.4)	68 (90.7)	0.190
**Surgical approach, *n*, %**				
Open	67 (32.7)	52 (40)	15 (20)	
Laparoscopy	138 (67.3)	78 (60)	60 (80)	**0**.**004**
**Primary tumor T stage, *n*, %**				
T1/T2	20 (9.8)	13 (10)	7 (9.3)	
T3/T4	160 (78.1)	100 (76.9)	60 (80)	0.827
Missing data	25 (12.1)	17 (13.1)	8 (10.7)	
**Primary tumor N stage, *n*, %**				
N0	77 (37.6)	48 (36.9)	29 (38.7)	Ref
N1	66 (32.2)	42 (32.3)	24 (32)	0.873
N2	35 (17.1)	22 (16.9)	13 (17.3)	0.958
Missing data	27 (13.1)	18 (13.9)	9 (12)	
**Primary tumor deposit, *n*, %**				
No	112 (54.6)	74 (56.9)	38 (50.7)	
Yes	62 (30.3)	36 (27.7)	26 (34.7)	0.292
Missing data	31 (15.1)	20 (15.4)	11 (14.6)	
**Primary tumor vascular invasion, *n*, %**				
No	113 (55.1)	74 (56.9)	39 (52)	
Yes	61 (29.8)	36 (27.7)	25 (33.3)	0.399
Missing data	31 (15.1)	20 (15.4)	11 (14.7)	
**Primary tumor perineural invasion, *n*, %**				
No	122 (59.5)	78 (60)	44 (58.7)	
Yes	52 (25.4)	32 (24.6)	20 (26.7)	0.764
Missing data	31 (15.1)	20 (15.4)	11 (14.6)	
**Primary tumor histologic grade, *n*, %**				
G1/2	154 (75.1)	100 (76.9)	54 (72)	
G3	43 (21)	27 (20.8)	16 (21.3)	0.795
Missing data	8 (3.9)	3 (2.3)	5 (6.7)	

Regarding the primary tumor characteristics, approximately two-thirds of patients presented with colon cancer (*n* = 129; 62.9%), and a small percentage presented with rectal cancer (*n* = 76; 37.1%). Synchronous liver metastases were present in the majority (*n* = 147; 71.7%), and metachronous liver metastases were present in only 28.3%. Of the primary tumor pathological stages, the majority of patients had T-stage 3 or 4 (*n* = 160; 78.1%), and half had N-stage N1 or N2 (*n* = 101; 49.3%). Less than one-third of patients showed positive results for vascular invasion of the primary tumor (*n* = 61; 29.8%), perineural invasion (*n* = 52; 25.4%) and cancer nodules (*n* = 62; 30.3%). There was a high percentage of G1 or G2 histological grading (*n* = 154; 75.1%).

In terms of tumor load, preoperative CEA was normal in a minority of patients (*n* = 56; 27.3%), while preoperative CA19-9 was normal in a majority of patients (*n* = 121; 59%). The number of metastases ≤2 was 126 (61.5%), and the maximum diameter of metastases was 33 ± 24 mm. Nearly half of the patients received neoadjuvant chemotherapy preoperatively (*n* = 89; 43.4%), and the remaining patients preferred direct surgery (*n* = 116; 56.6%). Almost all patients received adjuvant chemotherapy postoperatively (*n* = 192; 93.7%).

The percentage of laparoscopic surgery was higher in our center (*n* = 138; 67.3%) and slightly lower in open surgery (*n* = 67; 32.7%). Colorectal and liver surgery was performed in 132 cases (64.4%) at the same time, and colorectal surgery was performed first, followed by liver surgery in 73 cases (35.6%). The liver was resected anatomically in 40 cases (19.5%) and nonanatomically in 165 cases (80.5%). The duration of surgery was approximately 394.7 ± 165.2 min. As many as 153 (74.6%) of all patients had ASA scores of grade II.

### Clinical characteristics of patients based on cutting margin Status grouping

The R0 resection group and the R1 resection group were different in terms of type of hepatic resection, surgical approach, operative time, intraoperative bleeding, and neoadjuvant chemotherapy (Table 1). Specifically, patients who underwent anatomic resection were more likely to have R0 margins than those who underwent combined anatomic/wedge resection (*P* = 0.006) and wedge resection (*P* = 0.048). Laparoscopic surgery had a great minimally invasive advantage, but the proportion of R1 margins was higher than that of open surgery (*P* = 0.004). The R1 group had a longer operative time (*P* = 0.015) and more intraoperative bleeding (*P* = 0.023). The rate of R0 resection was higher in patients without neoadjuvant chemotherapy (*P* = 0.014).

### Survival analysis

**Figure 2:**
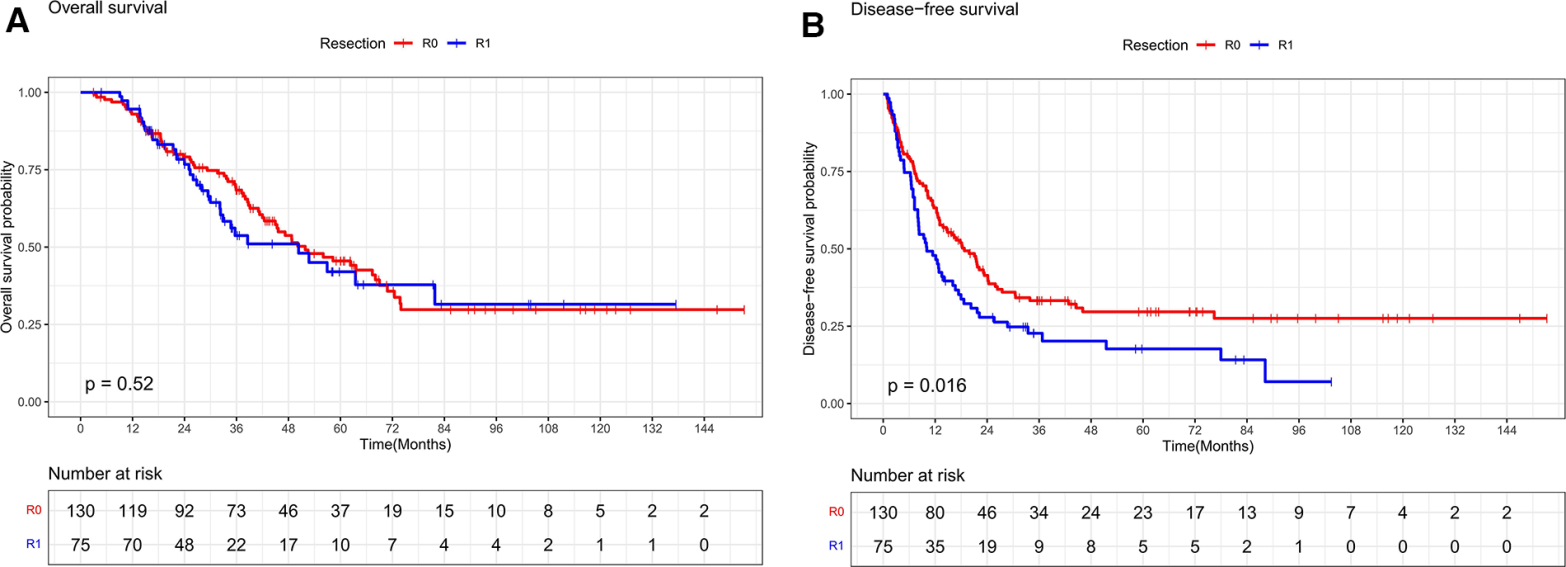
(**A**) Overall survival curves of R0 and R1 in CRLM patients (R0 ≥ 1 mm, R1 < 1 mm). (**B**) Disease-free survival curves of R0 and R1 in CRLM patients (R0 ≥ 1 mm, R1 < 1 mm).

The median follow-up time for the entire cohort was 60.1 months, with a median survival time of 50.1 months. The differences in overall survival rates of 93.4%, 68.4%, and 45.5% at 1, 3, and 5 years in the R0 resection group and 93.2%, 53.7%, and 42% in the R1 resection group were not statistically significant (*P* = 0.520, Figure 2A). The R0 resection group had significantly better disease-free survival rates of 63.2%, 33.3%, and 29.7% at 1, 3, and 5 years, respectively, than the R1 resection group (47.9%, 22.7%, and 17.7%, respectively) (*P* = 0.016, Figure 2B).

**Figure 3:**
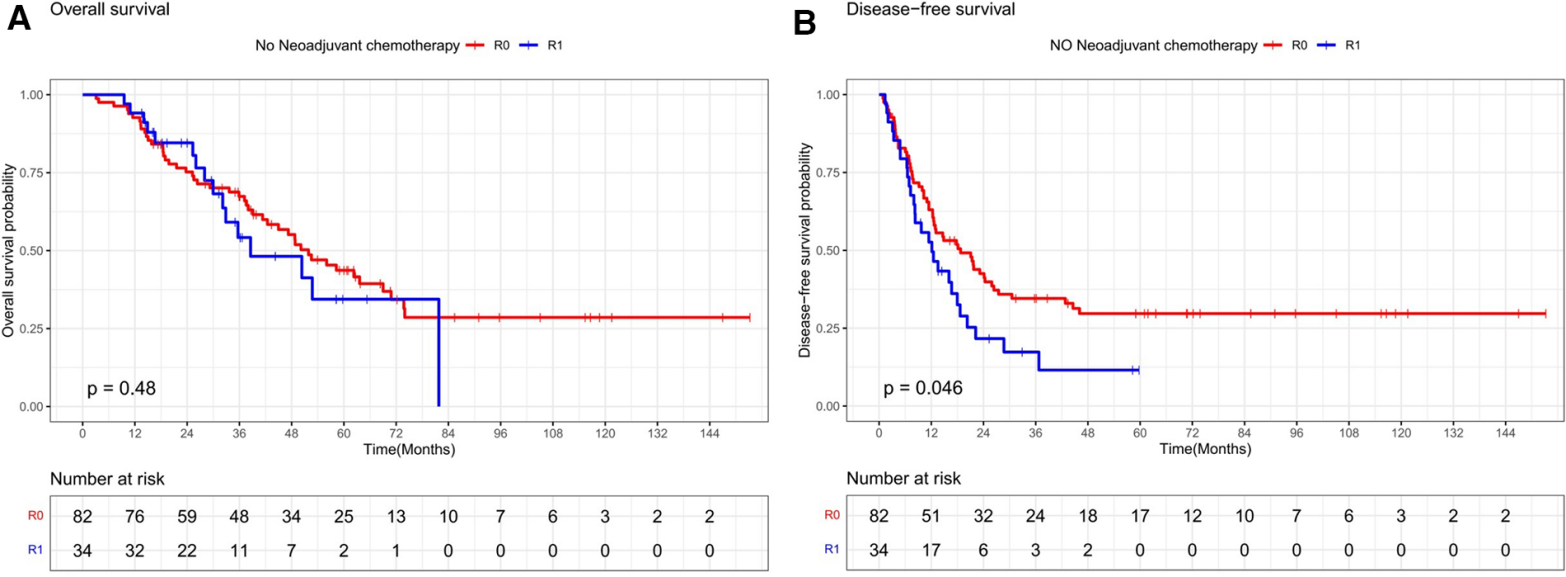
Os and DFS survival curves for R0 and R1 in patients not receiving neoadjuvant chemotherapy.

**Figure 4:**
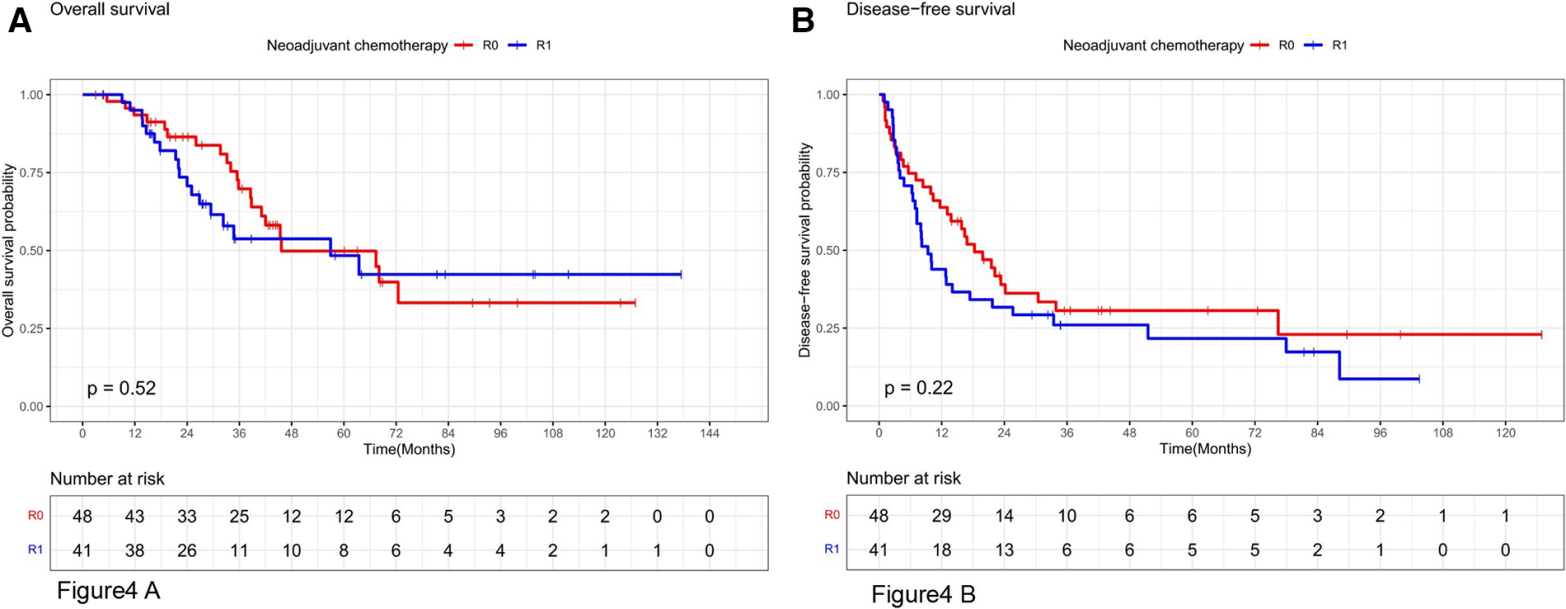
Os and DFS survival curves for R0 and R1 in patients receiving neoadjuvant chemotherapy.

Stratifying the neoadjuvant chemotherapy variables, the overall survival rates at 1, 3, and 5 years in the subgroup without neoadjuvant chemotherapy were 92.7%, 67.4%, and 43.7% in the R0 resection group and 92.3%, 54.2%, and 34.4% in the R1 resection group, respectively, which were not statistically significant (*P* = 0.48, Figure 3A). The disease-free survival rates at 1, 3, and 5 years in the R0 resection group were 63%, 34.6%, and 29.7%, respectively, which were better than 52.6%, 17.3%, and 11.6%, respectively, in the R1 resection group (*P* = 0.046, Figure 3B). The overall survival rates at 1, 3, and 5 years in the R0 resection group were 93.5%, 69.8%, and 49.9% vs. 92.5%, 53.8%, and 48.4% in the R1 resection group in the subgroup treated with neoadjuvant chemotherapy were not statistically significant (*P* = 0.52, Figure 4A). The difference in disease-free survival rates of 63.7%, 30.6%, and 23% at 1, 3, and 5 years in the R0 resection group vs. 43.9%, 26%, and 21.7% in the R1 resection group was not statistically significant (*P* = 0.22, Figure 4B).

### Defining independent risk factors for Os and DFS

The best cutoff values for the continuous variables in the cohort were selected using X-Tile software. Cox proportional risk regression analysis was used to determine the factors affecting overall survival. In the univariate analysis, operative time (HR = 2.17, *P* = 0.015), number of liver metastases (HR = 1.93, *P* = 0.001), maximum diameter of metastases (HR = 1.68, *P* = 0.021), preoperative CEA level (HR = 2.00, *P* = 0.005), preoperative CA19-9 level (HR = 2.58, *P* < 0.001), intraoperative bleeding (HR = 1.58, *P* = 0.036), surgical approach (HR = 0.65, *P* = 0.029), primary tumor N stage (N1: HR = 1.79, *P* = 0.02; N2: HR = 2.19, *P* = 0.005), primary tumor deposit (HR = 1.67, *P* = 0.02), primary tumor vascular invasion (HR = 1.66, *P* = 0.026), and primary tumor perineural invasion (HR = 1.63, *P* = 0.035) were significantly associated with the overall survival of CRLM patients. BMI (HR = 1.48, *P* = 0.057) approached statistical significance but was clinically significant. BMI and the abovementioned indicators were eventually included in the multivariable analysis. After adjusting for other competing risk factors, CA19-9 > 39 U/ml (HR = 2.29, 95% CI: 1.39–3.79, *P* = 0.001), primary tumor perineural invasion (HR = 1.78, 95% CI: 1.01–3.13, *P* = 0.047), and BMI > 24 kg/m^2^ (HR = 1.75, 95% CI: 1.05–2.93, *P* = 0.033) were independently associated with poor overall survival of patients (Table 2).

**Table 2:** Univariate and multivariable analysis of various clinical factors affecting the overall survival of the entire cohort.

	Univariate analysis			Multivariable analysis		
	HR	95%CI	*P* value	HR	95%CI	*P* value
**Liver margin**						
R0	Ref					
R1	1.15	0.76–1.74	0.520			
**Sex**						
Female	Ref					
Male	0.8	0.53–1.19	0.269			
**Age**						
<60	Ref					
≥60	1.1	0.74–1.63	0.645			
**BMI, kg/m2**						
≤24	Ref			Ref		
>24	1.48	0.99–2.2	**0** **.** **057**	1.75	1.05–2.93	**0**.**033**
**Surgery Interval**						
Staging surgery	Ref					
Simultaneous surgery	1.18	0.78–1.77	0.441			
**ASA score**						
I	Ref					
II	1.054	0.64–1.75	0.839			
III	0.542	0.35–1.73	0.542			
**Operative time, min**						
≤214	Ref			Ref		
>214	2.17	1.16–4.07	**0**.**015**	1.90	0.81–4.48	0.142
**Primary site**						
Colon	Ref					
Rectum	0.97	0.65–1.44	0.864			
**Timing of hepatic metastasis**						
Synchronous	Ref					
Metachronous	0.81	0.51–1.26	0.339			
**No. of liver metastases**						
≤2	Ref			Ref		
>2	1.93	1.31–2.86	**0**.**001**	1.33	0.79–2.26	0.277
**Size of largest tumour, mm**						
<50	Ref			Ref		
≥50	1.68	1.08–2.62	**0**.**021**	1.15	0.65–2.03	0.631
**Preoperative CEA level, U/ml**						
≤5	Ref			Ref		
>5	2.00	1.24–3.25	**0**.**005**	1.08	0.58–2.03	0.808
**Preoperative CA19-9 level, U/ml**						
≤39	Ref			Ref		
>39	2.58	1.73–3.83	**<0**.**001**	2.29	1.39–3.79	**0**.**001**
**Neoadjuvant chemotherapy**						
No	Ref					
Yes	0.87	0.59–1.3	0.51			
**Blood loss, ml**						
≤950	Ref			Ref		
>950	1.58	1.03–2.43	**0**.**036**	0.97	0.57–1.66	0.914
**Type of hepatic resection**						
Anatomical	Ref					
Anatomical + Wedge	0.99	0.57–1.74	0.981			
Wedge	0.79	0.49–1.27	0.323			
**Transfusion**						
No	Ref					
Yes	1.17	0.79–1.73	0.43			
**Postoperative chemotherapy**						
No	Ref					
Yes	0.75	0.36–1.55	0.435			
**Surgical approach**						
Open	Ref			Ref		
Laparoscopy	0.65	0.44–0.96	**0**.**029**	0.88	0.52–1.49	0.634
**Primary tumor T stage**						
T1/T2	Ref					
T3/T4	2.12	0.92–4.87	0.076			
**Primary tumor N stage**						
N0	Ref			Ref		
N1	1.79	1.1–2.92	**0**.**02**	1.00	0.55–1.80	0.988
N2	2.19	1.27–3.78	**0**.**005**	1.26	0.68–2.32	0.459
**Primary tumor deposit**						
No	Ref			Ref		
Yes	1.67	1.08–2.56	**0**.**02**	1.35	0.81–2.24	0.257
**Primary tumor vascular invasion**						
No	Ref		** **	Ref		
Yes	1.66	1.06–2.59	**0**.**026**	1.20	0.67–2.14	0.536
**Primary tumor perineural invasion**						
No	Ref		** **	Ref		
Yes	1.63	1.04–2.57	**0**.**035**	1.78	1.01–3.13	**0**.**047**
**Primary tumor histologic grade**						
G12	Ref					
G3	1.42	0.9–2.23	0.132			

The same approach was used to define the factors affecting disease-free survival. In the univariate analysis, liver margin (HR = 1.5, *P* = 0.016), BMI (HR = 1.41, *P* = 0.042), surgery interval (HR = 1.41, *P* = 0.05), operative time (HR = 1.82, *P* = 0.015), number of liver metastases (HR = 1.95, *P* < 0.001), maximum diameter of metastases (HR = 1.81, *P* = 0.002), preoperative CEA level (HR = 1.96, *P* = 0.001), preoperative CA19-9 level (HR = 1.58, *P* = 0.006), intraoperative bleeding (HR = 1.64, *P* = 0.007), T-stage of primary tumor (HR = 2.46, *P* = 0.009), and N-stage of primary tumor (N1: HR = 1.87, *P* = 0.002. N2: HR = 1.94, *P* = 0.006), primary tumor deposit (HR = 1.50, *P* = 0.03), primary tumor vascular invasion (HR = 1.98, *P* < 0.001), and primary tumor perineural invasion (HR = 1.70, *P* = 0.006) were significantly associated with disease-free survival in patients with CRLM. After controlling for all confounding factors, R1 margin (HR = 1.5, 95% CI: 1.03–2.19, *P* = 0.036) remained an independent risk factor for disease-free survival. In addition, the number of metastases >2 (HR = 1.65, 95% CI: 1.10–2.47, *P* = 0.016), the maximum diameter of metastases ≥50 mm (HR = 1.67, 95% CI: 1.06–2.64, *P* = 0.026), and vascular invasion of the primary tumor (HR = 1.65, 95% CI: 1.03–2.64, *P* = 0.038) were also independently associated with poorer disease-free survival (Table 3).

**Table 3:** Univariate and multivariable analysis of various clinical factors affecting the disease-free survival of the entire cohort.

	Univariate analysis			Multivariable analysis		
	HR	95%CI	*P* value	HR	95%CI	*P* value
**Liver margin**						
R0	Ref			Ref		
R1	1.5	1.08–2.09	**0** **.** **016**	1.5	1.03–2.19	**0**.**036**
**Sex**						
Female	Ref					
Male	0.97	0.69–1.37	0.878			
**Age**						
<60	Ref					
≥60	0.87	0.63–1.20	0.388			
**BMI, kg/m2**						
≤24	Ref			Ref		
>24	1.41	1.01–1.97	**0**.**042**	1.43	0.94–2.17	0.093
**Surgery Interval**						
Staging surgery	Ref		** **	Ref		
Simultaneous surgery	1.41	1.0–2.0	**0**.**05**	0.87	0.54–1.40	0.565
**ASA score**						
I	Ref					
II	1.23	0.77–1.97	0.381			
III	0.94	0.48–1.83	0.855			
**Operative time, min**						
≤214	Ref			Ref		
>214	1.82	1.12–2.95	**0**.**015**	1.31	0.66–2.58	0.438
**Primary site**						
Colon	Ref					
Rectum	0.89	0.64–1.25	0.511			
**Timing of hepatic metastasis**						
Synchronous	Ref					
Metachronous	0.86	0.72–1.04	0.126			
**No. of liver metastases**						
≤2	Ref			Ref		
>2	1.95	1.41–2.71	**<0**.**001**	1.65	1.10–2.47	**0**.**016**
**Size of largest tumour, mm**						
<50	Ref		** **	Ref		** **
≥50	1.81	1.24–2.65	**0**.**002**	1.67	1.06–2.64	**0**.**026**
**Preoperative CEA level, U/ml**						
≤5	Ref			Ref		
>5	1.96	1.32–2.92	**0**.**001**	1.40	0.84–2.34	0.196
**Preoperative CA19-9 level, U/ml**						
≤39	Ref			Ref		
>39	1.58	1.14–2.20	**0**.**006**	1.36	0.90–2.03	0.142
**Neoadjuvant chemotherapy**						
No	Ref					
Yes	1.11	0.80–1.53	0.549			
**Blood loss, ml**						
≤950	Ref			Ref		
>950	1.64	1.14–2.35	**0**.**007**	1.01	0.63–1.63	0.952
**Type of hepatic resection**						
Anatomical	Ref					
Anatomical + Wedge	1.52	0.94–2.47	0.089			
Wedge	0.88	0.57–1.35	0.557			
**Transfusion**						
No	Ref					
Yes	0.97	0.70–1.36	0.875			
**Postoperative chemotherapy**						
No	Ref					
Yes	1.74	0.77–3.95	0.184			
**Surgical approach**						
Open	Ref					
Laparoscopy	0.86	0.61–1.21	0.398			
**Primary tumor T stage**						
T1/T2	Ref			Ref		
T3/T4	2.46	1.25–4.84	**0**.**009**	1.55	0.76–3.17	0.232
**Primary tumor N stage**						
N0	Ref		** **	Ref		
N1	1.87	1.25–2.78	**0**.**002**	0.97	0.59–1.59	0.911
N2	1.94	1.21–3.13	**0**.**006**	1.18	0.68–2.06	0.548
**Primary tumor deposit**						
No	Ref			Ref		
Yes	1.50	1.04–2.16	**0**.**03**	1.09	0.71–1.66	0.700
**Primary tumor vascular invasion**						
No	Ref		** **	Ref		
Yes	1.98	1.38–2.85	**<0**.**001**	1.65	1.03–2.64	**0**.**038**
**Primary tumor perineural invasion**						
No	Ref		** **	Ref		
Yes	1.70	1.17–2.47	**0**.**006**	1.11	0.71–1.73	0.642
**Primary tumor histologic grade**						
G12	Ref					
G3	1.06	0.71–1.60	0.764			

### Nomogram of Os prognosis

**Figure 5:**
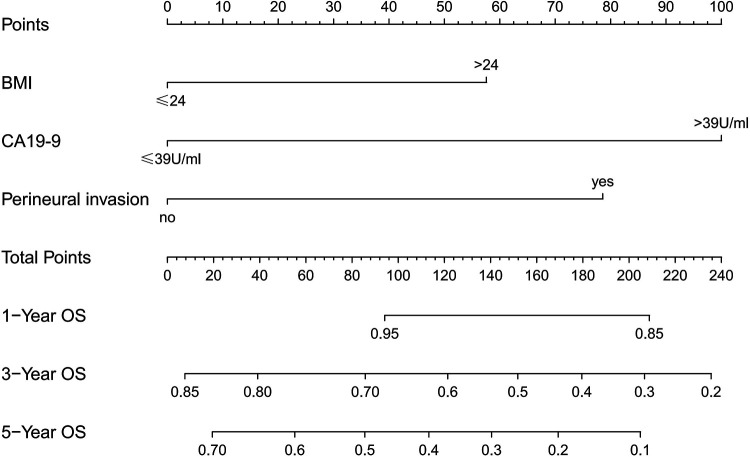
Nomogram predicting 1-, 3-, and 5-year overall survival in patients undergoing hepatectomy for CRLM.

**Figure 6:**
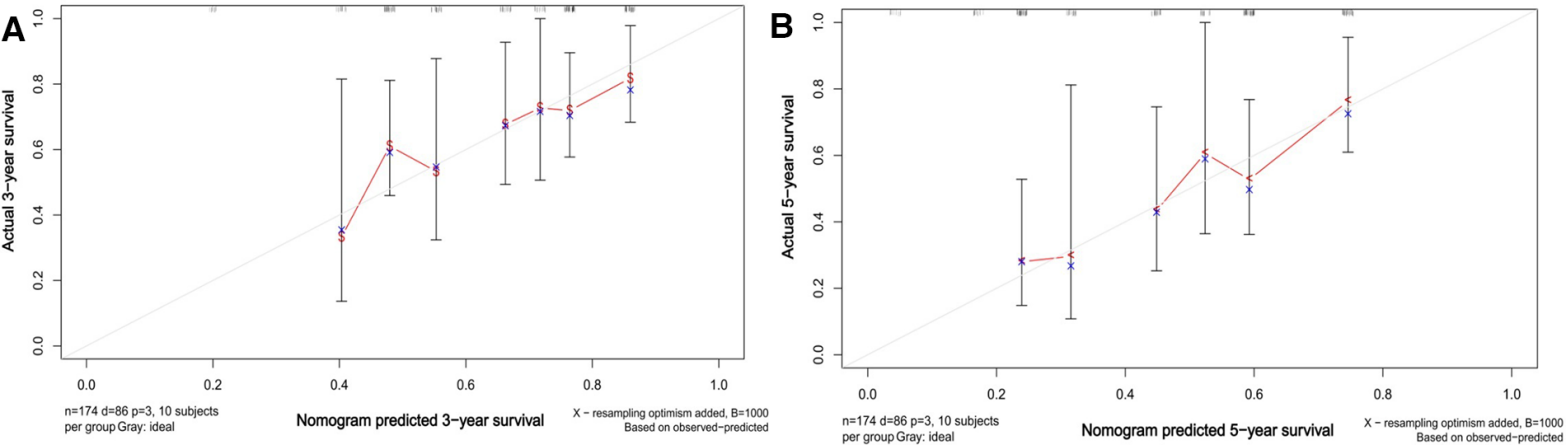
Calibration plots of the nomogram of OS predictions for CRLM hepatectomized patients. A and B show the predicted and actual 3- and 5-year survival probabilities for CRLM hepatectomized patients, respectively.

We created a nomogram containing the three factors mentioned above, thus enabling a more visual observation of the impact of each factor on prognosis (Figure 5). The calibration curve showed a good match between the actual and predicted probability of survival (Figure 6).

## Discussion

The most critical factor in achieving long-term survival in patients with CRLM is radical surgery, and the cutting margin is an essential focus. There have been conflicting opinions about the impact of cutting margins on the prognosis of patients with CRLM. First, the definition of the R1 cutting margin is different. As early as 1986, Ekberg et al. (14) reported the factors influencing the prognosis of liver metastases from colorectal cancer, where the extent of the tumor-free margin was the only treatment variable associated with survival time. Patients with tumor-free margins ≥10 mm (R0) had a significantly longer survival than those with margins <10 mm (R1). After that, everyone started to follow the “1 cm rule”. Both retrospective studies (15, 16) and prospective studies (17) have shown that the R1 (<10 mm) cut margin is an independent risk factor for patients with CRLM. Later, Fong Y et al. (18) first reported that there was no significant difference in prognosis between patients with negative cut margins but less than 1 cm and those with cut margins greater than 1 cm. Kokudo et al. (19) concluded that the minimum cut margin is not a significant prognostic factor affecting patient survival and that 2 mm can be considered the minimum clinically acceptable requirement. Pawlik TM et al. (20) analyzed the data of 557 patients with metastatic liver resection for colorectal cancer using a multicenter database and found no statistically significant 5-year survival and recurrence rates in three groups with margins of 1–4 mm, 5–9 mm and ≥1 cm, suggesting that a margin of less than 1 cm should not be a contraindication to surgical resection. In a prospective analysis of 293 patients, Hamady et al. (21) found no significant effect of 1-, 2-, 5- and 10-mm tumor-free margin widths on patient survival or recurrence rates. The awareness of the R1 cut margin is gradually narrowing. In 2015, the EGOSLIM (Expert Group on OncoSurgery management of Liver Metastases) group recommended a minimum acceptable margin of 1 mm for liver metastases from colorectal cancer (22).

However, even with the definition of R1 as a less than 1 mm margin, the prognostic impact of R1 remains highly controversial. Some studies concluded that R1 resection (tumor-free margin <1 mm) exhibited worse overall survival than R0 resection (tumor-free margin ≥1 mm) (23–25). However, different results were obtained in our study. Of the 205 patients included in our center, 130 (63.4%) were resected in R0, and 75 (36.6%) were resected in R1. OS at 1, 3, and 5 years was not significantly different between the R0 and R1 groups (*P* = 0.520), but DFS differed significantly between the two groups, with the R1 group being more susceptible to recurrence, and after correction for multifactorial analysis, the cut margin remained an independent risk for disease-free survival factor (*P* = 0.036). This is in agreement with that reported by Montalti R et al. (26). Although it has been shown that 50%–70% of intrahepatic microsatellite metastases are located within 1–2 mm from the tumor margin (27), the current electrosurgical devices used in liver resection, whether open or laparoscopic, can play an active role (28, 29). First, when liver tumors are removed with an electrotome, ultrasonic knife, or Cavitron Ultrasonic Surgical Aspirator (CUSA), some of the chopped liver tissue at the edge of the resection will be aspirated by suction. Second, after removal of the tumor, the surgical incision margin will be hemostatic with the application of energy instruments, which can cause cauterization coagulation necrosis of the tissue approximately 2–3 mm deep in the incision margin, achieving the same effect as RF ablation. Thus, even though some tumors may not have enough margin for resection, electrosurgical devices may destroy the remaining tumor cells. This may have caused some R0 cut margins to be incorrectly estimated as R1 cut margins, thus diminishing the difference between R0 and R1.

Other reports suggest that the widespread use of neoadjuvant chemotherapy and postoperative adjuvant chemotherapy has reduced the prognostic impact of the R1 margin, resulting in no significant difference in overall survival between R1 and R0 (30, 31). Margonis GA et al. (32) concluded that in the modern era of systemic chemotherapy, the impact of margin status on prognosis appears to be small compared to patient and tumor factors, and re-excision of R1 to R0 status does not improve long-term prognosis. However, some studies have given a different opinion and concluded that even with the addition of preoperative chemotherapy, it still does not change the outcome of R1 predicting poor outcome (33, 34). In this study, neoadjuvant chemotherapy was stratified for analysis, and among the 116 patients without neoadjuvant chemotherapy, R0 resection was 82 (70.7%) and R1 resection was 34 (29.3%). There was no difference in 1-year, 3-year, or 5-year OS between the two groups (*P* = 0.48), and the difference in DFS was statistically significant (*P* = 0.046). Of the 89 patients who received neoadjuvant chemotherapy, 48 (53.9%) were resected for R0 and 41 (46.1%) for R1. There was no difference in 1-year, 3-year, or 5-year OS (*P* = 0.52) or DFS (*P* = 0.22) between the two groups. The prognostic impact of R1 was not altered by neoadjuvant chemotherapy. The sample of only 13 patients without postoperative adjuvant chemotherapy in this study was too small to stratify, and therefore, it was not possible to assess whether the prognostic impact of R1 resection was influenced by postoperative adjuvant chemotherapy.

More interestingly, this study found that BMI >24 kg/m2 (HR = 1.75, 95% CI: 1.05–2.93, *P* = 0.033) was independently associated with poor overall survival in patients with CRLM. This has been scarcely reported in previous studies. Obesity is increasing worldwide, and it is a generally accepted view that it is a risk factor for the development of CRC (35). However, the relationship between overweight and the prognosis of patients with CRLM, especially after hepatectomy, is unclear. Meyerhardt JA et al. (36) reported that among women with stage II–III colon carcinoma, obesity (BMI ≥ 30 kg/m^2^) was associated with a significant increase in overall mortality (HR = 1.34, 95% CI: 1.07–1.67, *P* = 0.007). However, the mechanism of this correlation has not been fully determined. However, excessive obesity, especially high visceral fat content, is associated with insulin resistance and elevated insulin levels in the circulation. Raised insulin levels can contribute to increased circulating levels of insulin-like growth factor I (IGF-I), which promotes cell proliferation and inhibits apoptosis and is positively associated with the risk of colorectal cancer (37). However, such results have only been reported in female patients. In addition, obese patients may have more comorbidities at the same time, such as diabetes, hyperlipidemia, vasculopathy and other chronic diseases, which may cause more postoperative complications and indirectly affect the survival period. Although univariate analysis in this study found the association between BMI > 24 kg/m^2^ and prognosis to be close to statistically significant (*P* = 0.057), after adjusting for confounding factors, BMI > 24 kg/m^2^ was still found to be an independent prognostic factor for survival in patients with CRLM. This is something that has not been found in previous reports and is a focus of attention for surgeons.

The relationship between tumor markers and the prognosis of patients with CRLM is more frequently reported as CEA and prognosis (7, 26, 30). In our experience, CEA > 5 U/ml (HR = 2, 95% CI: 1.24–3.25, *P* = 0.005) and CA19-9 > 39 U/ml (HR = 2.58, 95% CI: 1.73–3.83, *P* < 0.001) in the univariate analysis were risk factors, but after adjusting for confounders, CA19-9 > 39 U/ml (HR = 2.29, 95% CI: 1.39–3.79, *P* = 0.001) remained an independent risk factor for OS. CA19-9 levels have been previously reported as a prognostic risk factor for patients with initially unresectable CRLM (38). The results of Sawada Y et al. (39) also suggest that high CA19-9 levels may reflect unfavorable tumor biology, especially in patients with advanced CRLM. Jiang LM et al. (40) followed up 85 patients who underwent liver resection combined with microwave ablation for CRLM and found that high CA19-9 levels were a poor prognostic factor for OS. Previously, there was a general focus on CEA and neglect of CA19-9. Our study is a reminder that CA19-9 is also a risk factor for 0S and should be taken seriously. A more detailed treatment plan should be developed for patients with high preoperative CA19-9 and intensive postoperative follow-up, thus achieving improved survival rates.

In addition to this study, primary tumor perineural invasion was established as an independent predictor of OS. Furthermore, a nomogram containing the above three factors was created to effectively and visually predict OS. This study included a very comprehensive set of study variables, so the final model showed good predictive performance.

In summary, this study established three independent risk factors for OS, however, the R1 margin was not included. This also suggests that the negative effect of the R1 margin should be downplayed. More attention should be paid to BMI > 24 kg/m2, and more in-depth studies are needed to explore the mechanisms of poor prognosis due to high BMI so that survival rates in this group can be improved in a targeted manner. And whether or not the CEA is normal, elevated CA19-9 should be a cause for concern and a more detailed treatment plan should be developed.

Our study also had some limitations. Firstly this is a retrospective study and the sample size of this study is small due to the limited number of patients in a single institution. Validation on prospective, multicentre and large-scale patients is necessary. After a rigorous and careful statistical analysis, we still obtained several risk factors for poor prognosis of liver metastases from colon cancer, and our statistical power is still good. Secondly almost all of our cohort had adjuvant chemotherapy administered postoperatively and it was not possible to assess whether the prognostic impact of the R1 margin was due to the adjuvant chemotherapy given postoperatively. Although some studies have drawn some conclusions (30)(, 32), further validation in populations not treated with adjuvant chemotherapy is still needed. This will allow a more accurate assessment of the role of the R1 margin. Finally, although this study included variables that produced confounding factors for analysis in the study as much as possible, the results of genetic examinations were not included in the study due to the large time span and the fact that there were some early patients who did not have genetic examinations; therefore, the effect of confounding factors of genetic examinations could not be eliminated (23).

## Conclusion

The negative effect of the R1 margin should be downplayed in patients undergoing liver resection for colorectal cancer liver metastases. Although the disease-free survival of the R1 margin is shorter than that of the R0 margin, it has no effect on the overall survival. The intraoperative preoccupation with the R0 resection margin at the expense of preserving the liver parenchyma is more than worth the cost. To improve overall survival, extra attention should be given to the factors of preoperative BMI, preoperative CA19-9, and the presence of perineural invasion of the primary tumor.

## Data Availability

The raw data supporting the conclusions of this article will be made available by the authors, without undue reservation.
